# “To Care for One Another on the Lands That Sustain Us”: Reflective Commentaries for Land-Based Healing Among Indigenous Cancer Survivors

**DOI:** 10.3390/ijerph23060740

**Published:** 2026-06-01

**Authors:** Hugh Burnam, Reesa R. Abrams, Marissa L. Bennett, Nancy Washburn, McKenzie Paterson, William O. Carson, Chelsea G. Redeye, Whitney Ann Henry, Josie Raphaelito, Rodney C. Haring

**Affiliations:** 1 Department of Indigenous Cancer Health, Roswell Park Comprehensive Cancer Center, Buffalo, NY 14263, USA; marissa.bennett@roswellpark.org (M.L.B.); nancy.washburn@roswellpark.org (N.W.); william.carson@roswellpark.org (W.O.C.); whitneyann.henry@roswellpark.org (W.A.H.); josie.raphaelito@roswellpark.org (J.R.); rodney.haring@roswellpark.org (R.C.H.); 2 Independent Researcher, Buffalo, NY 14261, USA; reesa.abr@gmail.com; 3 Independent Researcher, Washington, DC 20002, USA; fractaldesignstudios@gmail.com; 4 Department of Cancer Prevention and Control, Roswell Park Comprehensive Cancer Center, Buffalo, NY 14263, USA; chelsea.redeye@roswellpark.org

**Keywords:** Indigenous, American Indian, Alaska Native, Native Hawaiian, land-based healing, cancer survivorship, health, Haudenosaunee

## Abstract

**Highlights:**

**Public health relevance—How does this work relate to a public health issue?**

- This reflective commentary addresses how Indigenous cancer survivors in the United States experience significant gaps in culturally appropriate survivorship care.
- Histories of forced land removal, displacement, and broken treaties function as social determinants of health that continue to shape health outcomes for Indigenous Peoples.

**Public health significance—Why is this work of significance to public health?**

- This work provides perspectives of four Haudenosaunee People’s lived experiences and identifies traditional land-based healing practices as an important and currently absent component of Indigenous cancer survivorship programming.
- Traditional healing practices, Indigenous patient navigation, and land sovereignty are interconnected factors that current survivorship programs fail to address for AI/AN/NH communities.

**Public health implications—What are the key implications or messages for practitioners, policy makers, and/or researchers in public health?**

- Cancer survivorship programs for Indigenous Peoples must incorporate Indigenous patient navigation, family involvement, and traditional land-based healing practices that respect the cultural sovereignty and self-determination of Tribal Nations.
- Future programming and research must be built in partnership with Indigenous communities and guided by frameworks like the Relational Science Model that center reciprocity, humility, and respect.

**Abstract:**

Significant gaps exist in survivorship services across the cancer care continuum for Indigenous Peoples in the United States. Despite overcoming overwhelming cancer burden and high mortality risk, Indigenous cancer survivors report lower quality of life compared to non-Indigenous cancer survivors. Using an Indigenous social determinants of health framework, this article shares reflective commentaries from four Indigenous (Haudenosaunee) cancer care professionals who provide insights into the need for traditional Indigenous land-based healing practices among Indigenous cancer survivors, their families, and caregivers. Results suggest that (1) traditional Indigenous healing practices, (2) Indigenous patient navigation services, (3) communities of care, and (4) Indigenous lands and social determinants of health are important factors to support the health and wellbeing of Indigenous cancer survivors. Land-based healing for Indigenous cancer survivors requires further research for future implementation.

## 1. Introduction

For Indigenous cancer survivors globally, cancer survivorship programs are not commonly designed to meet the cultural priorities, community preferences, and individual needs for care [1]. In the United States, the National Institutes of Health (NIH) reports that 18.1 million people in the U.S. are cancer survivors (live with a history of a cancer diagnosis), yet significant healthcare gaps exist among American Indians (AI), Alaska Natives (AN), and Native Hawaiians (NH) which detrimentally impact their quality of life within the cancer care continuum [2,3,4]. Native populations have experienced a history of being diagnosed at earlier ages and at more advanced stages compared to other racial groups [5]. Additionally, after cancer treatment, Native Peoples experience the lowest 5-year cancer survival rates than any other racial population [6]. Despite overcoming overwhelming cancer burden and high mortality risk, Indigenous cancer survivors report lower quality of life compared to non-Native cancer survivors [7].

While dismal statistics on Indigenous cancer survivor rates exist, survivorship programs for Native Peoples continue to remain underfunded and under-resourced [6]. Cancer survivorship programs have long provided best practices such as standard exercise, nutrition counseling, psychosocial support, and clinical follow ups [8], but these practices lack cultural priorities for AI/AN and NH communities. Cancer burden continues to increase among AI/AN populations, including impact on the whole family, loss of income, family/caregiver relocating to care for their loved ones, and alterations in relationships. Survivorship services need to prioritize specific needs of AI/AN patients to include more personalized spiritual care, programs tailored to individual circumstances, increased involvement of family members, and connection to other Indigenous survivors.

The United States federal government has a legal federal trust responsibility to uphold the health and wellbeing of Native Peoples in their country [9]. AI/AN and NH experience higher rates of colorectal, breast, and cervical cancers than other racial groups [10]. In a comparison study between Native and non-Native cancer survivors’ quality of life (QOL), AI/AN scored lower on physical and social variations compared to non- AI/AN survivors, suggesting that there is a crucial need to support cultural practices in physical activity and incorporate more family and caregivers in AI/AN survivorship programs [5,7].

Since time immemorial, Indigenous lifeways have always been intrinsically connected to the land, yet systems of settler colonialism sought to erase those relationships and dispossess Indigenous Peoples of those lands. For generations, Indigenous Peoples have fought to maintain traditional practices, to collect foods and medicines, to carry forward original languages, to play medicine games which lift their spirits, and to continue to live in reciprocity. The land is not just a resource for Indigenous Peoples; it represents a physical, emotional, and spiritual connectedness which sustains life. From a global social determinants of health perspective, a history of forced removal of Indigenous Peoples from traditional lands and overall health decline are correlated [11,12].

Indigenous health practitioners recommend land-based healing approaches which incorporate modest physical activity (PA), family/caregivers, community support, and practice in local spiritual/cultural practices [13,14,15,16,17]. There are currently no reported data that reflect intentional use of land-based healing for Indigenous cancer survivors.

We came together as friends, colleagues, and community members to talk about Indigenous cancer survivorship. We shared stories with one another about how we have been impacted by cancer. We talked about being Indigenous and why the land is so important to us. In the months leading up to the development of this manuscript, we began conducting a scoping review about why the land is important for cancer survivors and their families. Our efforts were cut short when we noticed that there was a complete lack of research and programming around land-based healing for Indigenous cancer survivors. We felt compelled to share our experiences and how the land would be so integral to our healing, for our families, for our communities—and all of our Indigenous relatives who live with a cancer diagnosis.

We decided to write reflective commentaries from primarily Haudenosaunee perspectives. Roswell Parks Comprehensive Cancer Center is located on Haudenosaunee lands, specifically near three Seneca Nation territories and the Tuscarora Nation; therefore, we intentionally provide four Haudenosaunee-specific experiences to demonstrate our shared experiences within a local context. We felt that local perspectives were important for this manuscript and approach since we come from here. We can only speak of our personal experiences, and while we are not “representing” our specific nations in this article, nor speaking on behalf of our Haudenosaunee nations, we certainly represent ourselves as Haudenosaunee within a local context.

Important note: This is not a research project; it does not include a research question, nor research methods or design, and was not conducted on sovereign Indian lands. This is strictly a commentary and, as such, was not reviewable by an IRB.

### Use of Terminology

- “Cancer survivors” is used to mean people who live with a history of a cancer diagnosis.
- “Indigenous Peoples” is used interchangeably with “Native Peoples” or “Native” which we use to include American Indians (AI), Alaska Natives (AN), and Native Hawaiians (NH).
- “Racial groups” is used only for purposes of comparison among racially marginalized populations who also experience compounded inequities. Unlike many “racial groups” in the United States, AI/AN/NH hold inherent rights to sovereignty and self-determination as Native Nations.

## 2. Theoretical Framework

We use the World Health Organization’s (WHO) Social Determinants of Health (SDH) framework in Indigenous communities [18] to best examine health equity in Indigenous cancer survivorship, wherein healthcare providers must intentionally integrate place-based practices that honor the self-determination, cultural, and spiritual/holistic values of local Native communities. The WHO SDH framework for Indigenous communities emphasizes that Indigenous Nations have unique determinants, which may include culture, land, traditional practices, relationality, and stronger views on collectivism vs. individualism. Caroll et al. (2022, 5) explain that instead of a “linear, descriptive and prescriptive” Western conception of social determinants of health framework, use of Indigenous knowledge “uncovers the holistic network of interconnected determinants of health and well-being for indigenous nations” [18]. The WHO SDH framework in Indigenous communities seeks to examine and improve Indigenous Nation policies which support Indigenous sovereignty, self-determination, and community-based actions to strengthen culture, traditions, languages, and social ties. Indigenous land-based healing practices [19] suggest the use of culturally appropriate approaches for intergenerational wellbeing among Indigenous Peoples, which emphasize health sovereignty and are rooted in Indigenous knowledge for care.

Complementing this framework, the Relational Science Model [20] offers a practical structure for aligning these determinants with community-driven care by centering reciprocity, humility, respect, and integrity as foundational to any research or programing developed with Indigenous communities. Together, these frameworks emphasize that Indigenous cancer survivorship cannot be addressed with clinical approaches alone. Healing requires relational and land-centered processes that honor Indigenous Peoples and Nations.

## 3. Commentary Process of Development

Nine out of ten authors of this article are Indigenous. Four of the authors provided personal stories, anecdotes, and lived experiences to provide insights into Indigenous cancer survivorship and the need for land-based healing. Six of the other authors provided editing, revisions, and valuable feedback. This process was important for us because it reflects an approach often taken in our communities, an approach which encourages storytelling to compel further thought and consideration. These stories are often supported by other community members who feel as though particular individuals can speak to a topic based on their own experiences. In this case, the four authors were chosen by the group to represent land-based healing in cancer survivorship.

Reflective commentaries [21,22,23] can be helpful, especially to uncover impactful new directions for program considerations. As Indigenous scholars in cancer care services and programming, we echo similar sentiments as other Native scholars about Indigenous-based projects. In Windchief and Ryan, the authors wrote about Indigenous knowledge creation and collective identities by reflecting on their own experiences [24].

These stories reflect some shared experiences of Indigenous Peoples and also reflect what makes them unique as people and what gives them their identity while simultaneously serving to solidify relationships with others (2019, 353) [24].

Through storytelling, we endeavor to bring awareness to Indigenous survivorship programs and the need to address gaps in programming for Indigenous Peoples, especially as it pertains to the land and to our collective wellbeing. We use our own voices and experiences to share about approaches to cancer survivorship that prioritize Indigenous Ways of Knowing and approaches to healing that we have always known [25]. We now share our experiences in a way that is culturally appropriate for us as Haudenosaunee People, which we hope also opens pathways for future programming for Indigenous cancer survivors.

In the following section, we demonstrate differing perspectives between Reesa (Seneca, Allegany), Hugh (Mohawk, Akwesasne), Marissa (Seneca, Cattaraugus), and Nancy (Mohawk, Six Nations). Within these perspectives, Reesa and Hugh speak to land-based considerations and Marissa and Nancy speak to culture-based considerations for patients and survivors. These perspectives, we hope, offer insights into the need for both land-based healing and cultural approaches within cancer survivorship and across the cancer care continuum.

## 4. Results

### 4.1. Land Sovereignty as Health Equity: Reflections on Historical Trauma and Indigenous Cancer Survivorship (Reesa)

As a Seneca woman with a Master’s in Public Health who works for the Seneca Nation, I understand health not as an individual outcome, but as something fundamentally shaped by environment, relationships, and our community’s support. My work focuses on land use, community development, and systems-level planning. Through this work and my involvement with the Seneca Land Conservancy, a nonprofit working to return ancestral lands to Seneca stewardship, I have come to see that land sovereignty and health equity are inseparable. When Indigenous communities have control over their lands and can design spaces that reflect their values and needs, healing becomes possible in ways that conventional health systems cannot replicate.

I grew up on the Allegany Territory of the Seneca Nation, where I still live today. In the 1960s, a dam was constructed that removed approximately 600 individuals from treaty-protected land where Senecas had lived for generations. This dam directly impacted my community and many family members, including my father, grandmother, and extended family on my father’s side. The land we were removed from was protected by the Canandaigua Treaty, one of the oldest treaties between the United States government and Indigenous Nations. This territory itself represented only a fraction of our original land base, already reduced by centuries of forced removals and broken treaties. The construction of the Kinzua Dam was another act of forced relocation, breaking even this treaty-protected land agreement. It flooded our communities, our burial grounds, and the landscapes that had sustained us.

The Kinzua Dam represents a clear case study in how land loss translates directly to health impacts. While there has been limited formal research on the specific health outcomes of this forced relocation, the broader literature on historical trauma and forced displacement of Indigenous Peoples documents significant and lasting health consequences. We lost our land; we lost our traditional food systems, our cultural practices tied to place, and our social structures. As a result, we see to this day, increased rates of chronic disease, mental health challenges, and substance use.

My father was a teenager during the fight against the dam and throughout the forced relocation. During this time, he was drafted into the United States Army to fight in the Vietnam War. Upon returning, he experienced persecution for fighting in a war he had been forced into. He was removed from his land, forced to fight for a country that was not his own, and then blamed when he returned home. The compounding trauma of land loss and military service shaped his life in ways that are difficult to fully articulate. His experience illustrates how historical trauma operates at both individual and collective levels, shaping health outcomes across generations.

I have also witnessed cancer’s impact within my own family, though I respect their privacy in not sharing details here. What I observed was the relentless cycle of appointments, the weight of navigating health systems, the toll on both patient and caregivers, and the rare moments of peace found in simply being outside together. These observations, combined with my understanding of historical trauma and my work on land sovereignty, have led me to consider how land-based healing could support Indigenous cancer patients and their families.

My work with the Seneca Land Conservancy has reinforced that land back is not just about territory. It is about restoring our ability to care for the land and to be taken care of by it. From a public health perspective, this means recognizing land access as a social determinant of health. Land sovereignty is health sovereignty.

In my community work, I created a women’s hiking group where we gather, move, and spend time outdoors together. It is a space to be present without needing to share too much but still healing together, individually and collectively. I have watched women arrive carrying the weight of daily life and leave lighter. Seeing this has reinforced my understanding of land as medicine, as a teacher, and as part of collective healing. Spending time outdoors and moving through the land has been central to how I maintain wellbeing and process the intergenerational impacts of displacement. Movement through the land offers a rhythm that helps settle the nervous system.

From a public health perspective, the barriers Indigenous cancer patients face are well documented: geographic isolation, lack of culturally appropriate care, mistrust of medical systems rooted in histories of harm, and economic constraints. For Indigenous communities, these barriers are compounded by historical trauma from ongoing forced removal, broken treaties, and continuing struggles for land sovereignty and self-governance. Land-based healing programs could address some of these barriers by meeting people where they are, both literally and figuratively.

I imagine cancer patients having intentional time on the land with their families, not as another appointment or obligation, but as respite. A chance to sit outside together, to walk at whatever pace feels right, and to remember who they are beyond their diagnosis. For Indigenous cancer survivors whose communities have experienced forced displacement, returning to the land carries additional meaning. It is a reclamation of what was taken. Creating the infrastructure for land-based healing requires policy changes that support land back initiatives and recognition that access to ancestral lands is a matter of health equity. Health is not solely an individual responsibility, but a collective outcome shaped by relationships, place, and our ability to care for one another and the lands that sustain us.

### 4.2. I Want Them to Spend Time Together: Indigenous Cancer Survivorship, Identity, and the Land (Hugh)

“Remember that hike that we did in the Adirondacks? Did Dad like that hike?” I asked my mom. “Yeah, I do remember, and yes, he liked it. He liked it because we were all together doing something,” she said.

We were recalling a hike that we had all done together as a family over three years ago now. I can recall his laughter, and I hear his voice, as we trekked through the woods in the Adirondack mountains. This was just over a year before my dad’s cancer diagnosis.

It was October of 2022, and my family took a trip to the Adirondack Mountains in Upstate New York. We all went hiking on a path which led to Cobblestone Lookout. Our family went on this hike: my youngest son (Dash), my mom (Lana) and dad (Mark), my two brothers (Tyler and Brad), my sister (Cheyenne), her boyfriend (Russ), my oldest niece (Rynn), and me. The fall leaves were scattered through the trails, our boots crunched the leaves on the trail, and I remember joking with everyone—our laughter echoed through the woods. We saw different animals and plants, and the birds flew above. We arrived at the “lookout” portion of the trail and just sat there in awe. The sun shone across the mountains, casting shadows in the valleys. We sat there in moments of silence. It felt important to me. The next year, in the fall of 2023, we came back to hike again. My sister’s boyfriend proposed to her—our dad was so proud. I have so many great memories of my family in the Adirondacks. It is, after all, traditional lands of the Mohawk and the Algonquin Peoples.

In the spring of 2024, months after our last hike there, our dad was diagnosed with stage 4 non-Hodgkin’s lymphoma, specifically diffuse large B-cell lymphoma (DLBCL), a blood cancer. Our family was devastated by the news of his diagnosis. In the ensuing months, our dad visited many different doctors, and he went through several tests and aggressive treatments, which included chemo and radiation therapy. He was exhausted physically and mentally, but he was driven to be with his family. During this time, he visited his friends and family members more. He was given medicinal teas and extracts made by our traditional healers in the community to ease pain, and he talked a lot about lacrosse, which is a medicine game to us. It was evident that traditional Indigenous healing practices were important to him as a Mohawk (Haudenosaunee) person. In late October of 2024, after several rounds of CAR-T cell treatment (chimeric antigen receptor t-cell therapy), our dad went into remission. The signs and symptoms of cancer were gone. Immediately, I thought about being together and doing something as a family again. I wanted to bring them to the mountains, to celebrate. It was, to me, the closest thing that reminded me of my childhood—all of us together.

I Googled light exercises for cancer survivors and health benefits. I looked at land-based healing projects. I thought about our lands as Indigenous Peoples and how beneficial land-based healing and traditional medicines could be to patients and their families. Our dad always liked to talk about that type of stuff. I found plenty about cancer survivorship and even some about light exercises including hiking and walking programs for survivors. But, there was almost nothing about Indigenous cancer survivors and land-based healing practices.

This is the hard part. Our dad was only in remission for a handful of months when the cancer relapsed and came back. This time, it was even more aggressive. My family was completely shocked, and we scrambled to help him. Our mom was his rock and so important for him as he went back into treatment, where this time, the burden of the disease felt heavier on him, on her, and on us. We raised money for his treatment, rallied our friends and family. Our dad even opened up to us more about his life and about how he was so proud of us. His vulnerability felt strange, his words felt soft, and he was more open. He spoke with care in his heart, even more than he typically would.

Our dad was a world-renown lacrosse player and coach. He coached the Haudenosaunee Nationals lacrosse team. He helped countless Native kids get into college as student athletes. They all came together for him during this time. My dad’s brothers arranged for lacrosse coaches from across the country to visit him over Zoom. Many of his friends called him, and he appreciated their presence. We enjoyed seeing him smile and laugh with his friends. He talked about spirituality, about his Catholic faith. Every morning at 6 am, he would receive a text from his friend who was also a cancer survivor; his friend provided a daily quote from the Bible. Our community organized a medicine game for him—it was the last lacrosse game that he would see.

Our mom was by his side the whole time, the rock of our little family; she shouldered the weight of his diagnosis, and she showed us why she was the backbone of our family. Our dad referred to her as “his angel” who was sent to him by God. I thought: The love they have for each other is so incredibly rare.

I imagined him healing. I imagined him spending time with us. I thought about those times in the mountains. I thought about my two sons (Hugh and Dash), my nieces (Rynn and Laela), and my unborn nephew (Red) spending time with him. I went through a range of emotions for months; we all did. We held him close. My brothers, my sister, my mom, and I held onto him as he took his final breaths. My sister held him for hours after his passing. Her daughters and husband stood by her. I held my brothers close. My mom lost her best friend. We lost our superhero. He passed away on 10 September 2025.

In our grief, we remember him and his smile. We remembered the lasting impressions he left, not just on us, but on his extended family, his friends, and all the people across the Haudenosaunee Confederacy and around the world. He was well loved. The memory of my dad inspires my interests in hiking and land-based healing for Indigenous cancer survivors. I want them to have time with their loved ones in a special place, together. I want them to have what I imagined for my family, had my dad survived. I want them to spend time together, like we did.

### 4.3. Community Support Systems Are Critical for Indigenous Cancer Survivors (Marissa)

Through my work as an Indigenous oncology patient navigator, I have encountered countless Indigenous cancer patients and survivors. As a member of the community I work with and through my schooling in community health and Native American leadership, I have seen and continue to see many barriers to care and factors that affect treatment outcomes and survivorship of Indigenous People.

Historical trauma and mistrust of the medical system often prevent Indigenous People from participating in cancer screenings at recommended ages. One of my main roles in patient navigation is making patients feel comfortable and secure in the healthcare system. This includes educating about the importance of early detection and cancer screening recommendations, being a trusted support person, and advocating in their cancer journey. I meet patients at the cancer center as their first appointment is usually nerve wracking and anxiety-inducing, showing them where to go in the cancer center and attending the appointment with them if they would like me to. Often, patients appreciate this extra support as they can have a trusted person to debrief with after the appointment or an extra set of ears to catch any details they may have missed and ensure they receive culturally competent care. I am a member of the Seneca Nation, and I work with Indigenous cancer patients, which I feel builds an extra layer of trust in our interactions.

Social determinants of health such as economic instability, social and community connections, and geography play huge roles in patients accessing cancer treatment and taking part in survivorship programs. In my role as a patient navigator, most of my work is addressing these barriers by coordinating transportation, looking for financial assistance programs, providing education about screening and treatment, and facilitating monthly support groups.

In my experience working as a patient navigator, support systems including family and community are a positive and important factor to treatment and survivorship outcomes. I facilitate a monthly support group for Indigenous cancer patients and survivors. Although the group is co-led by me and a community health nurse from our Nation’s health center, the group is community-driven as it was started at the request of community members. Membership ranges from newly diagnosed patients to survivors close to their five-year survivorship anniversary.

The group allows for a safe space for sharing and connecting about the unique experience of being an Indigenous person with cancer navigating the healthcare system. Members share advice about coping with treatment physically, emotionally, and spiritually. They are also able to share their journey while in treatment without fear or mistrust about their personal medical information being shared. Members of the group build connections and support people they can lean on in their own community when they are feeling overwhelmed or discouraged about their cancer journey.

Seeing the positive effects of support and survivorship programs like the support group, I believe Indigenous cancer survivors would strongly benefit from additional programs that offer them a sense of community and connection with other survivors. Including family in these programs would offer even more benefits to the survivor and their family. In my conversations with patients and survivors, family always comes up, or their family is often right there with them every step of the way. Actively engaging family in survivorship programs would provide them with a positive outlet and time to connect with the survivor. Cancer is stressful for both the patient and family, so being able to participate in programs like land-based healing would allow for survivors and family to engage in healthy and positive time together after the stress and pressure that is cancer treatment.

### 4.4. Indigenous Cancer Survivorship: Self-Reflection (Nancy)

3 October 2022 is a day that changed the path I would walk moving forward. At just 37 years old, I was diagnosed with ductal carcinoma in situ (DCIS), a non-invasive form of breast cancer, in my left breast. As a wife, a mother of three, and an Indigenous oncology patient navigator at the time, I was wrapped in love and support from family, friends, and colleagues through my entire cancer journey. I am now three years cancer-free, a time that has reshaped my view on life and the passion I have in the work that I do.

During the diagnosis and treatment phase of my journey, much of my healing was turned to my traditions as an onkwehonwe woman. I used traditional medicine as protection and grounded myself through my beadwork, in combination with surgical Western medicine and genetic counseling/screening. Thankfully, the only treatment necessary was surgical; I opted to do a total bi-lateral mastectomy. Though DCIS is non-invasive, I chose this course of treatment due to the extensive amount of calcification clusters in my imaging, due to the high-grade cells found during biopsies, and to reduce the worry of recurrence that I knew I would carry. I went through a total of four surgeries, three of which were reconstructive, over a course of one year. Within this time, I hit a mental brick wall that had me doubting myself as a wife and as a mother because of how I now looked. It took almost a full year and all four surgeries before I felt comfortable in my own skin again. As for my mental health, I turned to counseling through a provider at my cancer center; I grounded myself through beading. During this time, my son was in his lacrosse season. Lacrosse for Haudenosaunee people is medicine, and I took full advantage of that medicine and found myself healing while watching my boy play the game he loves.

My work as an Indigenous patient navigator was also greatly impacted by my cancer journey. I returned to work 10 weeks after my first surgery and continued to work through the remaining three surgeries, taking minimal time off as possible. As I returned to work, I took some time reflecting on how I would share this experience with my co-workers, communities, and patients I served. The way I approached my work moving forward shifted from being an Indigenous patient navigator to a survivor who is an Indigenous patient navigator. As new patients came in, I found myself relating to that first initial fear after a diagnosis, and my approach to assisting all the patients I navigated became gentler.

After about a year and a half I decided I wanted to share my story a bit more broadly. In many Indigenous communities across the globe, cancer is not a disease that is heavily discussed, as some believe when it is talked about, it is then welcomed. Because of this, I decided to approach sharing my experience with caution and instead invited those who wanted to hear. Working with colleagues, we were able to bring my story to a local Indigenous community, where I was able to engage in Q&A and provide insight into cancer screenings and stigma that may go along with it in Indigenous communities. From this education session, I was approached by many community members who thanked me for encouraging them to get screened and asked for contacts for genetic counseling. To this day, I am still approached by Indigenous community members for resources, questions, and just an ear to listen when it comes to cancer experience within our communities.

## 5. Discussion

This reflective commentary offers four (4) themed categories in which Indigenous cancer survivors and their families may benefit from traditional Indigenous land-based healing practices.

### 5.1. Traditional Indigenous Healing Practices

Since time immemorial, Indigenous-based healing practices have existed. All commentaries described traditional healing practices as rooted in the land and cultural practices of the survivors. Nancy and Hugh expressed the importance of the medicine game (lacrosse) to help lift the minds of Indigenous cancer survivors. Reesa and Hugh described the importance of hiking on the land to spend time together and to reconnect with Indigenous expressions of culture and worldview. Nancy explained that traditional Indigenous medicines can be used with Western medicine for treatment and healing purposes. These traditional practices reflect Indigenous knowledges, which ensure community-based interactions designed to support a sense of self-determination and to build trust. Reesa shared that the women’s hiking groups she created helped build individual and group wellness:

“Seeing this has reinforced my understanding of land as medicine, as a teacher, and as part of collective healing. Spending time outdoors and moving through the land has been central to how I maintain wellbeing and process the intergenerational impacts of displacement.”

Among Indigenous Peoples, traditional land-based practices contribute directly to the healing journey.

In the article authored by Johnson-Jennings, Billiot, and Walters, “Indigenous individuals consider the land as co-contributing to their meaning of place and influencing their healing journey. Land then serves as an active agent in healing, provides a culturally appropriate location to conduct healing, and supports the common factors of healing found in most health interventions around the world” (2020, 4) [26]. Traditional Indigenous practices in gathering plant medicines [17], traditional medicine games [27], and human/non-human relationships [28,29] with the natural world are rooted in the land, designed to support physical, mental, and emotional healing [30]. Land-based healing practices are shown to improve health outcomes among cancer survivors, including activities like hiking, walking, canoeing, and other non-strenuous activities [31,32,33,34], yet studies do not specifically describe traditional healing practices of Indigenous Peoples. For Native Hawaiians, ʻāina (land) can be an indicator of health [35]. Keli‘iholokai et al. describe further land-based healing connected to the land:

ʻāina and a connection to ʻāina is a key solution to health and addressing health concerns that have stemmed from colonization and social injustices… Reconnecting with ancestral land and cultural practices has been shown to address health inequities and promote resilience among other Indigenous communities.

(2020, 10) [36]

Traditional healing practices on the land are central to resiliency and wellbeing of Indigenous Peoples, where once, traditional ways of life were always accessible to everyone.

### 5.2. Communities of Care

Across all of the commentaries, the inclusion of family, community, time with other cancer survivors, and opportunities to share about cancer survivorship creates supportive and empowering environments for Indigenous cancer survivors. According to Cavanagh et al., “[I]indigenous survivors may benefit from programs which share stories of other indigenous survivors who have successfully overcome cancer” (2016, 339) [1]. Nancy describes the importance of reflecting about her experience as a survivor and sharing her story with the larger community to ensure Indigenous communities see what survivorship looks like and receive education about cancer screening.

Marissa stated that “Including family in survivorship programs would provide them with a positive outlet and time to connect with the survivor… land-based healing would allow for survivors and family to engage in healthy and positive time together after the stress and pressure that is cancer treatment.” Physical activity and familial support among Indigenous cancer survivors have been shown to be considerably effective for Indigenous cancer survivors [14]. Hugh and Reesa both mentioned spending time with community and story sharing as such a crucial element to healing. A more holistic approach to Indigenous cancer survivorship care involves the emotional connection to relationships. Gifford et al. describe this connection further, “The physical domain involves taking care of one’s body; the mental involves rational thought; the emotional encompasses relationships and being connected to family and community; and the spiritual involves beliefs, values, and identity” (2020, 7032) [37], including relationships (relationality) with humans and non-human relatives for resilience [28].

### 5.3. Indigenous Cancer Patient Navigation

Marissa and Nancy both described the importance of patient navigation and advocacy across the cancer care continuum. Early screening, diagnosis, community education, patient advocacy, access to services, coping with treatment, and community connections are critical to care for community members, cancer patients, and survivors. A history of mistrust exists between Indigenous communities and medical institutions. Marissa and Nancy’s reflections suggest that to address those complex histories, community members need advocates who reflect their population. Marissa states that patients need to feel as comfortable as possible as they receive services. Indigenous patient navigation is housed within the Department of Indigenous Cancer Health at Roswell Park Comprehensive Cancer Center. According to Henry et al., the goal of Indigenous patient navigation is partnership between Indigenous Nations and health systems, “[Partnering] helps to create the trust needed to proactively provide early screening and detection information and train eligible community members to provide cancer and cancer-screening education” [4]. Indigenous patient navigators are specifically trained in cancer-focused work with Indigenous and rural population as a service program (which is not tied to research or clinical trial recruitment). Indigenous patient navigators support Indigenous Peoples across the cancer care continuum, from prevention to survivorship.

### 5.4. Indigenous Lands and Social Determinants of Health

Indigenous identity is commonly rooted in the land. Reesa described that land-based healing offers to cancer patients the opportunity to “remember who they are beyond their diagnosis”, which addresses a longstanding stigma. The land, she describes, offers space of “respite” and care for Indigenous cancer survivors. Indigenous lands, like Indigenous Peoples, have been subject to systems of injustices and settler colonialism. Returning to the land for purposes of health equity, movement, medicine, and re-connection brings Indigenous cancer survivors important reminders about their unique relationship to the land.

In recognition of the social determinants of health for Indigenous Peoples, Reesa writes that land sovereignty is health sovereignty. Self-determination and health resonates with Caroll et al.

Unique determinants are particular to each Indigenous Nation, such as culture, use of natural resources for health and healing, traditional practices and ceremonies, and language… reclaiming Indigenous health begins with community-driven, Nation-based processes grounding in sovereignty and self-determination.

(2022, 8) [18]

Indigenous cancer survivors should be able to choose the best possible care for them- selves, for their families, and for their communities. Applying the Relational Science Model [20] here means that any land-based healing initiative must be developed with Indigenous communities, grounding the program design with humility and respect for the sovereignty and self-determination of each Nation. Cancer survivors are provided the space to share their experience and knowledge within a communal space designed to empower and heal, with Indigenous sovereignty and self-determination as central to any given project for survivorship care.

## 6. Recommendations

This project advocates for expanded services for Indigenous cancer survivors to engage in new and unconventional programming specific to land-based healing, grounded in traditional Indigenous healing practices which have existed since time immemorial. While land-based healing approaches may offer benefits to cancer survivors broadly, these practices carry a specific cultural, spiritual, and historical weight for Indigenous Peoples that cannot be generalized. Given the disproportionate cancer burden experienced by AI/AN/NH communities, compounded by histories of forced displacement, broken treaties, and systemic health inequalities, land-based healing for Indigenous cancer survivors is not simply a wellness preference but an act of cultural continuity and health sovereignty.

Cancer survivorship programs are often overlooked across the cancer care continuum. The Department of Indigenous Cancer Health at Roswell Park Comprehensive Cancer Center promotes the Indigenous & Rural Patient Navigation Program, which was created to approach Indigenous communities for cancer center partnership [9]. Patient navigators who are active in the community they serve have a greater impact of reducing barriers to cancer care for their patients, including survivorship. Patient navigators are often the first line of communication for patients who have mistrust in the healthcare system and will be more willing to partake in a palliative care nature therapy related to survivorship. Survivorship programs need to embed patient navigation into programming, and for Tribal Nations, and rural and urban Indigenous populations, respect for Indigenous sovereignty is vital in program services [9,38].

Programs to improve the quality of life for cancer survivors are often underfunded across the United States [39]. Tribal Nations and Native Hawaiians across the United States have an inherent right to healthcare services, and the United States should continue to fund important services that incorporate the unique needs of Indigenous Peoples [40]. Prioritizing enhanced cancer survivorship services aimed at advancing health equity among Indigenous Peoples is an active practice of acknowledging the federal trust responsibility of the United States government to provide healthcare to Tribal Nations. Native Hawaiians hold federal rights established under the Native Hawaiian Health Care Act of 1988 [41]. Through recognition of Indigenous self-determination, hospitals can continue to rebuild trust with Indigenous Peoples. Hospital policy makers, healthcare researchers, community members, and Tribal leadership must continue to recognize Indigenous sovereignty to provide important healthcare services. We emphasize the need for Indigenous workforce and Indigenous scholars for future programming and works due to the nuances needed and the importance of lived experiences and the specific context of the many Tribal Nations.

Unique considerations must be made for Indigenous cancer survivors, rooted in extended family care, land-based healing, and traditional Indigenous knowledge. Cancer patients, survivors, and their caregivers experience the burden of cancer in complex ways. Extended family involvement, community, and nationhood play vital roles for Indigenous cancer survivors and in their journey towards healing. Survivorship services should also provide spaces for increased opportunities to share their experiences with a larger community for advocacy and education. Traditional Indigenous healing practices, including traditional land-based healing practices, are vital to the wellbeing of Indigenous Peoples.

The Relational Science Model [20] offers a practical framework for aligning medical research practice with Indigenous communities of care by prioritizing reciprocity, humility, respect, and integrity throughout the research process, within the context of community-driven services. In practice, this means survivorship program developers must prioritize Indigenous community partnership at every stage, ensuring that land-based healing initiatives reflect the values, needs, and sovereignty of the Nations they serve rather than replicating extractive research models of the past.

## 7. Conclusions

This reflective commentary utilized four personal narratives from Indigenous members of the Department of Indigenous Cancer Health at Roswell Park Comprehensive Cancer Center. These stories centered around the themes of (1) traditional Indigenous healing practices, (2) communities of care, (3) Indigenous cancer patient navigation, and (4) Indigenous lands and social determinants of health. Our authors’ narratives emphasize the unique challenges and experiences of Indigenous People, specifically, the Haudenosaunee, as well as general experiences of cancer survivors, their families, and their communities. This examination highlights the importance of cancer centers focused on Indigenous health issues and the need for greater funding and recognition for Indigenous ways of healing in cancer survivorship and across the cancer care continuum.

## Data Availability

No new data were created or analyzed in this study. Data sharing is not applicable to this article.

## Abbreviations

AI	American Indians
AN	Alaska Natives
NH	Native Hawaiians
